# Efficient estimation of bounded gradient-drift diffusion models for affect on CPU and GPU

**DOI:** 10.3758/s13428-021-01674-7

**Published:** 2021-09-24

**Authors:** Tim Loossens, Kristof Meers, Niels Vanhasbroeck, Nil Anarat, Stijn Verdonck, Francis Tuerlinckx

**Affiliations:** KU LEUVEN, Tiensestraat 102 - bus 3713, 3000 Leuven, Belgium

**Keywords:** Affect dynamics, Affective Ising Model, CPU, Euler-Maruyama, GPU, Metropolis-Hastings, Non-linear diffusion models

## Abstract

Computational modeling plays an important role in a gamut of research fields. In affect research, continuous-time stochastic models are becoming increasingly popular. Recently, a non-linear, continuous-time, stochastic model has been introduced for affect dynamics, called the Affective Ising Model (AIM). The drawback of non-linear models like the AIM is that they generally come with serious computational challenges for parameter estimation and related statistical analyses. The likelihood function of the AIM does not have a closed form expression. Consequently, simulation based or numerical methods have to be considered in order to evaluate the likelihood function. Additionally, the likelihood function can have multiple local minima. Consequently, a global optimization heuristic is required and such heuristics generally require a large number of likelihood function evaluations. In this paper, a Julia software package is introduced that is dedicated to fitting the AIM. The package includes an implementation of a numeric algorithm for fast computations of the likelihood function, which can be run both on graphics processing units (GPU) and central processing units (CPU). The numerical method introduced in this paper is compared to the more traditional Euler-Maruyama method for solving stochastic differential equations. Furthermore, the estimation software is tested by means of a recovery study and estimation times are reported for benchmarks that were run on several computing devices (two different GPUs and three different CPUs). According to these results, a single parameter estimation can be obtained in less than thirty seconds using a mainstream NVIDIA GPU.

## Introduction

Continuous-time stochastic modeling is becoming increasingly popular in the field of affect research (de Haan-Rietdijk et al.,, 2017; Hamaker et al.,, 2015; Oud, 2002, 2010; Voelkle & Oud, 2013). Continuous-time models, like the Ornstein-Uhlenbeck (OU) model (de Haan-Rietdijk et al.,, 2017; Hamaker et al.,, 2015; Oud, 2002, 2010; Voelkle & Oud, 2013) and the Affective Ising Model (AIM; Loossens et al., 2020) have several advantages over discrete-time models, such as the popular vector autoregressive model (Bos et al., 2012; Bringmann et al., 2013; Lodewyckx et al., 2011; Pe et al., 2015; Snippe et al., 2015; Wichers, 2014; Zheng et al., 2013); for instance, affect processes are generally conceived as continuously unfolding across time. A continuous-time description is more conform with this idea. By modeling affect processes as if they evolve continuously, issues with unequally spaced time intervals are naturally resolved. Unequally spaced time intervals are common in affect studies, especially studies concerned with affect dynamics in daily life which generally rely on experience sampling methods (Bolger et al., 2003, 2013). Although studies could be designed where time intervals between observations are equally spaced, unequal intervals are often desirable for the ecological validity of the data (Hektner et al., 2006) or for increasing the efficiency by which information about the process being studied can be obtained (Voelkle & Oud, 2013).

Another aspect of affect dynamics that is brought more frequently into the spotlight is its non-linear nature. Evidence has been found for heavily skewed (Crawford & Henry, 2004; Merz et al., 2013; Yik et al., 1999), V-shaped (Kuppens et al., 2013; Mattek et al., 2017) and even multimodal data distributions (Loossens et al., 2020). There is a particular interest in non-linear dynamics because of possible relationships with chaotic behavior and catastrophe theory (Van der Maas & Molenaar, 1992). Claims have been made, for instance, about autocorrelations and cross-correlations increasing before a switch to a state of depression – a signature which is credited to the effects of critical slowing down before a phase transition (Leemput et al., 2014). Non-linearities, like the presence of multiple attractors, could also describe regime-switching dynamics, a dynamical feature that is believed to be an important characteristic of certain psychopatologies of affect, such as bipolar disorder (Hamaker et al., 2015) and borderline personality disorder (Houben et al., 2016).

Although non-linear dynamics have caught the attention of affect researchers, continuous-time models that incorporate and unify such dynamics and whose parameters can also be estimated on real data remain scarce. Serious attempts are nonetheless being made to promote continuous-time modeling (Driver et al., 2017). Software has been developed for parameter estimation of the OU model – the continuous-time variant of the lag-1 vector autoregressive model. Possibilities for a state space formulation of the model or a hierarchical formulation have also been made available. The development of performant software for parameter estimation and related analyses is crucial because unlike for the popular discrete-time autoregressive models, closed-form expressions for the parameters estimates of continuous-time stochastic models, such as the OU model, generally do not exist. One has to rely on numerical, and (global) optimization schemes in order to locate the global optimum of the likelihood function. Optimization algorithms often require numerous objective function evaluations in order to locate the global optimum which can constitute a computational bottleneck. Although the OU model does not have closed-form expressions for its parameter estimates, it does have a closed-form expression for its likelihood function. Such a closed form expression is beneficial when a large number of function evaluations are required. For non-linear models, even the likelihood function itself need not have a closed-form expression; in that case, one has to rely on simulation-based or numerical approximations to obtain the likelihood function, making the optimization procedure more complicated and computationally expensive.

Recently, the Affective Ising Model (AIM) was introduced, which is a non-linear, continuous-time, stochastic model for affect (Loossens et al., 2020). The model was applied to two-dimensional affect data characterised by the positive (PA) and negative affect (NA) dimensions. It was demonstrated that the AIM is able to capture non-linearities that commonly occur in experience sampling data, like skew, V-shape and bimodalities. Similar to the OU model, fitting of the AIM is done using maximum-likelihood optimization. Likelihoods are preferably approximated numerically using graphics processing units (GPU), but approximations can also be obtained using Central Processing Units (CPU). The min-log-likelihood function is minimized using a global optimization heuristic: differential evolution (DE; storn & Prive 1997).

In this paper, we introduce a Julia (https://Julialang.org) software package dedicated to fitting the Affective Ising Model. Julia is a performant scientific programming language that includes packages for writing GPU code. Furthermore, it is open and free to use. First, we will briefly discuss the AIM as introduced by Loossens et al., (2020). Next, we will discuss the numerical algorithm that is used in the software package to compute the likelihood of the model. Subsequently, we elaborate on the structure of the software package and the different options that are included. The package includes an implementation of the DE heuristic for the minimization of the min-log-likelihood function. The min-log-likelihood function can be numerically computed using either a GPU or a CPU. In the final section of the paper, we discuss the accuracy of the numerical approximation method by comparing it to the traditional Euler-Maruyama approximation scheme. We also test the estimation software by means of a recovery study. Additionally, some benchmark estimations have been included to demonstrate the performance of the estimation software both on GPU and CPU for different computing devices (a standard ‘thin and light’ laptop, a workstation laptop and a workstation desktop).

## The Affective Ising Model

the Affective Ising Model (Loossens et al., 2020) describes the evolution of the momentary experienced levels of positive and negative affect, which we will denote as *y*_1_(*t*) and *y*_2_(*t*) respectively. In the absence of positive sensations, *y*_1_ equals zero. Similarly, *y*_2_ equals zero in the absence of negative sensations. If the momentary experienced positive affect is overwhelming, on the other hand, *y*_1_ is equal to one. Likewise, *y*_2_ equals one when the momentary negative affect is overwhelming. Thus, 0 ≤ *y*_*i*_ ≤ 1 for *i* = 1,2.

According to the AIM, the dynamics of the affect state **y**(*t*) = (*y*_1_(*t*),*y*_2_(*t*)) is governed by the stochastic differential equations

1 $$ \mathrm{d} y_{i}(t) = - D \frac{\partial F(\mathbf{y}(t))}{\partial y_{i}}  \mathrm{d}t + \sqrt{2D}  \mathrm{d}W_{i}(t), $$

with *i* = 1,2. The function *F*(**y**) depicts the free energy of the affect system, which is unique to every individual. It includes all interactions involved in orchestrating affective experiences. From a modeling perspective, it can be considered a potential surface on which the affect state **y**(*t*) evolves. The parameter *D* is known as the diffusion constant and is also unique to every individual. It determines how quickly the affect state **y**(*t*) evolves on the potential surface *F*(**y**) – it is a time-scaling parameter. Smaller values make time move slower (for the same amount of time, the system will change less), while larger values make time move faster (for the same amount of time, the system will change more). The functions *W*_*i*_ denote uncorrelated Wiener processes, introducing inherent stochasticity to the system.

The free energy function *F*(**y**) of the AIM is given by

2 $$ F(\mathbf{y}) = \sum\limits_{i = 1}^{2} \bigg[ -{{\varLambda}}_{i} {y_{i}^{2}} + {{\varTheta}}_{i} y_{i} + N_{i} \big(y_{i} \ln(y_{i}) + (1-y_{i}) \ln(1-y_{i}) \big) \bigg] + {{\varLambda}}_{12} y_{12}. $$

The parameters ${{\varLambda }}_{i} \in \mathbb {R}^{+}$ represent positive feedback parameters; both the positive and negative affect systems are self-exciting. The parameter ${{\varLambda }}_{12} \in \mathbb {R}$ depicts the mutual interaction between the positive and negative dimensions. When *Λ*_12_ > 0, the subsystems are mutually inhibiting; strong positive sensations will suppress negative sensations and vice versa. When *Λ*_12_ < 0, the subsystems are mutually exciting; positive sensations will stir negative sensations and the other way around. The parameters ${{\varTheta }}_{i} \in \mathbb {R}^{+}$ depict thresholds. The larger the threshold, the more difficult it is for the corresponding dimension to become agitated. The terms involving the parameters *N*_*i*_ ≥ 0 and the logarithms are related to the entropy of the affect system. Underlying the positive and negative affect systems are actually two pools containing an incredible number of stochastic binary units (see Loossens et al., 2020). These units are an abstract representation of all neuronal, hormonal and chemical processes involved in brain and body to establish affect. Given this underlying system, the entropy describes what affect states **y** are more probable and which are less probable should there be no interactions. By virtue of the interactions, affect states that would rarely be experienced because of the entropy can become more natural. For a more elaborate description of the parameters and their interpretation, see Loossens et al., (2020).

Equation 1 describes how the affect system updates in an infinitesimal interval d*t*. Aggregating all these small changes (i.e., integrating the equation over time), one can determine the position of the affect state given the underlying model parameters and a previous observation **y**(*t*_0_). However, because of the inherent stochasticity, it is impossible to determine the position of the affect state exactly. There are many pathways the affect system could have taken since the previous observation. At most, the conditional probability of the affect state being somewhere in the affect space – the unit square – can be determined.

**Fig. 1:**
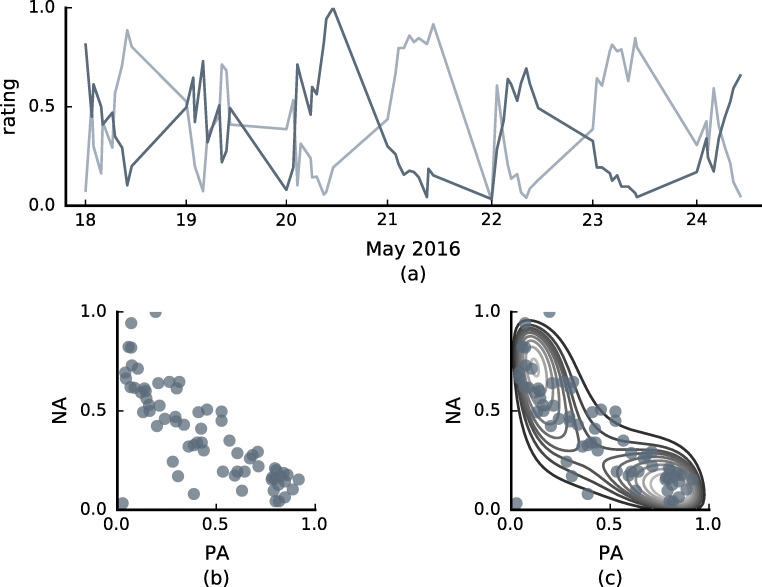
Illustration of ESM data **a** A time series of PA (light grey) and NA (dark grey) ratings. This is an extract from the data discussed by Heininga et al., (2019). **b** A scatter plot of the data from panel (a). **c** The same scatter plot as in panel (b), but combined with a fit of the AIM. The contour depicts the altitude lines of the equilibrium distribution of the AIM fit

There are two effects that influence the conditional probability: drift and diffusion. The term in Eq. 1 involving the d*t* is called the drift term. It governs the deterministic evolution of the affect state. The affect state is bound to drift towards local minima of the free energy function, even if external fluctuations were absent. Because of the logarithmic terms in the free energy, the drift term is non-linear. By virtue of the non-linearity, the free energy function of the AIM can have multiple local minima (up to four in a two-dimensional space). The presence of multiple local minima will result in multimodal data patters, since the affect state is more likely to be found around local minima (for an illustration of a bimodal AIM fit, see Fig. 1c or see Loossens et al., 2020). In addition, the combination of the non-linearity and the mutual inhibition between the PA and NA dimension can result in V-shaped, nonlinear correlations between PA and NA as often observed in real data (Larsen et al., 2017; Kuppens et al., 2013; Mattek et al., 2017). Heavily skewed data distributions can also be generated.

The term in Eq. 1 containing the Wiener processes is the source of the inherent stochasticity or diffusion effect. Although the affect state is bound to drift toward local minima, part of the change in the affect state is probabilistic – a random fluctuation. On an infinitesimal timescale, the Wiener processes are Gaussian distributed with mean zero and variance d*t* (see e.g., (Gardiner, 1986)). The Wiener processes are uncorrelated. In addition, they are also uncorrelated in time.

If we were to prepare someone in a specific affect state and let him evolve for a while, over and over again, always for the same period time, we would always get different outcomes because of the inherent stochasticity. Were we to do this an infinite number of times, we would however notice that there are specific regions in the affect space where the person ends up most of the time, while other regions remain largely unexplored. After the evolution has initiated, there is a probability density function *p*(*t*,**y**) that specifies where the affect state is more probable to be observed and where it is not. This probability density depends on the initial state (where it started), the model parameters, and the time that has elapsed. The more time has elapsed, the more ground could have been covered.

The evolution of the conditional probability density function *p*(*t*,**y**) is governed by the Fokker-Planck equation,

3 $$ \frac{\partial p(t,\mathbf{y})}{\partial t} = D \sum\limits_{i = 1}^{2} \bigg\{ \frac{\partial}{\partial y_{i}}\bigg(\frac{\partial F(\mathbf{y})}{\partial y_{i}} p(t,\mathbf{y}) \bigg) + \frac{\partial^{2}p(t,\mathbf{y})}{\partial {y_{i}^{2}}} \bigg\} $$

with initial condition

4 $$ p(0,\mathbf{y}) = \delta(\mathbf{y} - \mathbf{y}_{0}). $$

Since all possible emotional experiences live in the unit square, the affect state can never cross the boundary of this space. We therefore also have to impose the no-flux boundary conditions (see Appendix A)

5 $$ \bigg\{ \frac{\partial F(\mathbf{y})}{\partial y_{i}} p(t,\mathbf{y}) + \frac{\partial p(t,\mathbf{y})}{\partial y_{i}} \bigg\} \bigg\vert_{\partial {{\varOmega}}} = 0, $$

where *∂Ω* denotes the boundary of the unit square. The initial condition (4) states that we let the affect state evolve from a known position **y**(0) = (*y*_1,0_,*y*_2,0_) at time *t* = 0. Here, the time of the last measurement is considered to be at *t* = 0 and **y**(0) simply denotes the last observation. The boundary condition (5) states that the affect state cannot cross the boundary, the probability flux through the boundary is zero (but there can be probability mass *on* the boundary). The first term on the right-hand side of Eq. 3 describes the effect of the drift on the conditional probability density function. The second term is a pure diffusion term.

Because the affect state is attracted toward local minima of the free energy function and because of the boundedness of the affect space, the conditional probability density *p*(*t*,**y**) stabilizes in the long-time limit – it becomes stationary. The stationary distribution is given by the Boltzmann distribution

6 $$ p(\mathbf{y}) = \lim_{t\rightarrow \infty}p(t,\mathbf{y}) = \frac{e^{-F(\mathbf{y})}}{Z}, $$

where *Z* denotes the normalization constant,

$$ Z = {{\int}_{0}^{1}}\mathrm{d}\mathbf{y}  e^{-F(\mathbf{y})}. $$

If enough data is collected over a sufficiently long period of time, the Boltzmann distribution (6) will be the same as the distribution of the data (provided the data was generated by AIM mechanisms). The stationary distribution specifies what regions of the affect space are more likely to be visited by an individual and what regions are unlikely to be visited, unconditional on any previous state.

Many models encountered in affect psychology, which are primarily related to the Ornstein-Uhlenbeck (OU) model (de Haan-Rietdijk et al.,, 2017; Oud, 2002, 2010; Voelkle and Oud, 2013), have a description similar to the AIM. However, they often do not have any nonlinear terms in the drift nor are they bounded. As a consequence, their conditional and stationary distributions are Gaussian (see (Loossens et al., 2020)). For the AIM, both the conditional and stationary distributions deviate from Gaussianity.

## Data

Although the AIM has an abstract (neuro) physiological inspiration (Loossens et al., 2020), it was introduced as a model for affective time series obtained via experience sampling (ESM) (Csikszentmihalyi & Larson, 1987; Hektner et al., 2006) and daily diary (Bolger et al., 2003) methods. In general ESM and daily diary studies, participants are instructed to carry around a mobile device (typically a smartphone or palmtop) in their daily life. Throughout the day, or once at the end of the day, they fill out several surveys about their momentary emotion levels. Participants rate the intensity of a fixed set of discrete emotion items (e.g., *How happy do you feel at the moment?*), usually on a Likert scale or a continuous scale ranging from *not at all* to *very much*. Capturing people’s emotions (almost) in the moment has the strength that it not only significantly reduces memory biases, the ambulatory nature of these assessments also yields emotional time series that are high in ecological validity (Dejonckheere et al., 2019). If positive and negative affect are not explicitly included as items in the surveys, PA and NA ratings at a given time point are generally constructed by averaging over the positive and negative affect ratings, respectively, obtained at that time.

An illustration of a typical ESM time series is depicted in Fig. 1a. Positive affect ratings are depicted in lighter grey and negative affect ratings are depicted in darker grey. In Fig. 1b, a scatter plot of the same data is shown. In Fig. 1c, the same scatter plot is shown, but in combination with a fit of the AIM. The contour lines depict the equilibrium distribution of the model fit. We can see that the equilibrium distribution has two distinct modi.

## Estimation

The AIM includes eight parameters **Θ** = {*Λ*_1_,*Λ*_2_,*Λ*_12_, *Θ*_1_,*Θ*_2_,*N*_1_,*N*_2_,*D*}. Given a time series {**x**_*j*_ = (*x*_1*j*_,*x*_2*j*_)|*j* = 0,1,...,*n* − 1} of momentary PA and NA ratings, these parameters can be estimated using maximum-likelihood optimization. In order to make the affect ratings interpretable for the model, they have to be transformed so that they fall between 0 and 1. We then identify each (transformed) measurement **x**_*j*_ with a position in the affect space:

$$ \mathbf{x}_{j} = \mathbf{y}(t_{j}). $$

### The likelihood function

Parameter estimates $\hat {\boldsymbol {{{\varTheta }}}}$ can be found by maximizing the likelihood function. The likelihood function ${\mathscr{L}}(\boldsymbol {{{\varTheta }}} \vert \{ \mathbf {x}_{j} \}^{j = 0, ..., n-1})$ of the parameter set **Θ** given the data {**x**_*j*_|*j* = 0,1,...,*n* − 1} is a product of the likelihoods of the individual data points:

$$ \mathscr{L}(\boldsymbol{{{\varTheta}}}  \vert  \{ \mathbf{x}_{j} \}^{j = 0, ..., n-1} ) = \ell(\boldsymbol{{{\varTheta}}}  \vert  \mathbf{x}_{0})  \prod\limits_{j = 1}^{n-2}  \ell(\boldsymbol{{{\varTheta}}}  \vert  \mathbf{x}_{j}, \mathbf{x}_{j+1}), $$

where the likelihoods *ℓ*(**Θ**|**x**_*j*_,**x**_*j*+ 1_) of the parameter set **Θ** given the consecutive measurements (**x**_*j*_,**x**_*j*+ 1_) are defined as

$$ \ell(\boldsymbol{{{\varTheta}}}  \vert  \mathbf{x}_{j}, \mathbf{x}_{j+1}) = p_{\boldsymbol{{{\varTheta}}}}(t_{j+1}, \mathbf{x}_{j+1}  \vert  t_{j}, \mathbf{x}_{j}). $$

Here, *p*_**Θ**_(*t*_*j*+ 1_,**y**|*t*_*j*_,**x**_*j*_) corresponds to the probability density function of **y** conditional on the observation **x**_*j*_ = **y**(*t*_*j*_) at time *t*_*j*_ and the AIM parameters **Θ**. It is a solution of the Fokker-Planck (3) with **Θ** as parameters and initial condition

$$ p(t_{j},\mathbf{y}) = \delta(\mathbf{y} - \mathbf{x}_{j}). $$

The initial data point **x**_0_ cannot be evaluated conditionally on some prior information because there is no information of the affect state prior to this observation. If this data point is assumed not to be exceptional in any way, it should probably lie within a likely region in the affect space, which can be determined using the stationary distribution (6). In that case, the likelihood *ℓ*(**Θ**|**x**_0_) of the parameter set **Θ** given the initial measurement **x**_0_ is given by

$$ \ell(\boldsymbol{{{\varTheta}}}  \vert  \mathbf{x}_{0}) = p_{\boldsymbol{{{\varTheta}}}}(\mathbf{x}_{0}), $$

where *p*_**Θ**_(**y**) denotes the stationary distribution of **y** corresponding to the parameter set **Θ**. If there is however reason to believe something exceptional has occurred before the initial measurement, something which might have disrupted the affect state in such a way that it is possible that **x**_0_ does not nicely fall within the stationary distribution, then it is better to ignore this data point in the optimization of the likelihood and use **x**_0_ only to condition on for the likelihood contribution of **x**_1_. In practice, this can be done by setting *ℓ*(**Θ**|**x**_0_) equal to 1.

In general, it is not the likelihood function that is maximized, but the min-log-likelihood function which is minimized. The min-log-likelihood function is defined as the negative logarithm of the likelihood function,

$$ \begin{array}{@{}rcl@{}} \Tilde{\mathscr{L}}(\boldsymbol{{{\varTheta}}}  \vert  \{ \mathbf{x}_{j} \}^{j = 0, ..., n-1} ) &=& - \ln(\mathscr{L}(\boldsymbol{{{\varTheta}}}  \vert  \{ \mathbf{x}_{j} \}^{j = 0, ..., n-1} )) \\ &=& -\ln(\ell(\boldsymbol{{{\varTheta}}}  \vert  \mathbf{x}_{0})) - \sum\limits_{j = 1}^{n-2}  \ln(\ell(\boldsymbol{{{\varTheta}}}  \vert  \mathbf{x}_{j}, \mathbf{x}_{j+1})). \end{array} $$

### Numerical solution

There does not exist a closed-form expression for the conditional probability densities *p*_**Θ**_ that make up the likelihood function – an analytic solution for the Fokker-Planck (3) of the AIM is unavailable. We therefore have to rely on numerical methods to construct the min-log-likelihood function.

Using Eq. 1, we could simulate numerous trajectories using the Euler-Maruyama algorithm (see, e.g., kloeden & Platen 1992), all starting from the same initial state **y**(*t*_*j*_) = **x**_*j*_ and using a specific discrete time step *Δt*. If we let them all evolve for a time *t*_*j*+ 1_ − *t*_*j*_, the distribution of the endpoints will approximate the conditional probability density function *p*_**Θ**_(*t*_*j*+ 1_,**y**|*t*_*j*_,**x**_*j*_) (upon proper normalization). Although this method is fairly straightforward and the computation of a single trajectory has got a low computation cost, a tremendous number of trajectories have to be simulated in order to obtain a smooth approximation and an additional kernel smoothing function may be required (see, e.g., Verdonck et al.,^2016^). When the numeric approximations are obtained by means of such stochastic simulation procedures, the randomness is passed on to the min-log-likelihood. In other words, computing the min-log-likelihood twice, we will not end up with the same result. This makes it difficult to determine the parameter set $\hat {\boldsymbol {{{\varTheta }}}}$ that minimizes the min-log-likelihood function since the result will inherently vary, unless a sufficient number of trajectories are simulated to obtain smooth solutions for the min-log-likelihood functions.

Another issue with the Euler-Maruyama algorithm is that we have to evaluate the partial derivatives *∂*_*i*_*F*(**y**) in Eq. 1 each time we want to update the system (*∂*_*i*_ is a shorthand notation for $\frac {\partial }{\partial y_{i}}$). Because of the logarithmic terms in the expression of the free energy (2), its partial derivatives diverge toward infinity on the boundary *∂Ω* of the affect space – positive or negative, depending on which side. This indicates that the free energy function is very steep at the boundary. In the neighborhood of this steep “wall”, appropriate measures have to be taken in order to control the size of the simulated steps and avoid overshooting. Since the partial derivatives of the free energy appear in combination with the step size in Eq. 1, controlling the step size essentially comes down to choosing a sufficiently small time step. The smaller the simulation step, the more steps have to be simulated to bridge a specific period of time. As such, simulating even a single trajectory can become time consuming.

Instead of simulating trajectories, we can also numerically solve the Fokker-Planck (3). The advantage of solving the Fokker-Planck equation is that we can obtain smooth solutions without any randomness. The Fokker-Planck equation (3) is a linear, second-order partial differential equation. It can be rewritten as

$$ \begin{array}{@{}rcl@{}} \frac{\partial p(t,\mathbf{y})}{\partial t} &=& D \sum\limits_{i = 1}^{2} \bigg\{ \frac{\partial^{2} F(\mathbf{y})}{\partial {y_{i}^{2}}} + \frac{\partial F(\mathbf{y})}{\partial y_{i}} \frac{\partial}{\partial y_{i}} + \frac{\partial^{2}}{\partial {y_{i}^{2}}} \bigg\} p(t,\mathbf{y}) \\ &=& L p(t,\mathbf{y}), \end{array} $$

where *L* represents the linear differential operator. We can discretize the affect space so that we obtain an equally spaced grid with spacing *δ*. Because the partial derivative of the free energy is singular on the boundary, we position the grid cells at the corners of the grid in such a manner that their centers coincide with the points $\left (\frac {\delta }{2}, \frac {\delta }{2}\right )$, $(\frac {\delta }{2}, 1-\frac {\delta }{2})$, $(1-\frac {\delta }{2}, \frac {\delta }{2})$ and $\left (1-\frac {\delta }{2}, 1-\frac {\delta }{2}\right )$. By discretizing the affect space, the application of the differential operator *L* can be approximated as a matrix vector-multiplication, describing how the probability density *p*_*mn*_(*t*) in each of the cells $\left (\frac {2m+1}{2}\delta ,\frac {2n+1}{2}\delta \right )$ changes over an infinitesimal time interval *Δt* (see, e.g., Chang & Cooper 1970). Applying these changes to each of the cells, an approximation of the conditional probability density *p*(*t* + *Δt*,**y**) at time *t* + *Δt* can be obtained. Repeating this procedure *k* times, we obtain the approximate solution at time *t* + *kΔt*. In the remainder of the text, we will refer to the cell $\left (\frac {2m+1}{2}\delta ,\frac {2n+1}{2}\delta \right )$ using the short-hand notation (*m*,*n*).

The matrix approximation of *L* can be constructed by approximating the partial derivatives *∂*_*i*_*p*(*t*,**y**) and ${\partial ^{2}_{i}}p(t,\mathbf {y})$ using finite differences (see e.g., Chang & Cooper 1970). However, the operator *L* contains the partial derivatives *∂*_*i*_*F*(**y**) and ${\partial _{i}^{2}}F(\mathbf {y})$, which are both singular at the boundary where they diverge to infinity. Just like for the Euler-Maruyama method, this implies that the step size *Δt* and the bin width *δ* have to be chosen sufficiently small in order to obtain meaningful approximations. Smaller step sizes *Δt* means having to do more updates to bridge a specific time interval. Also, smaller bin widths *δ* give rise to more cells that have to be updated each time step.

Instead of relying on finite differences, we will use a Metropolis-Hastings’ updating scheme to describe the effect of the differential operator *L* (see e.g., Kikuchi et al., 1991). Consider the cell (*m*,*n*) with probability density *p*_*mn*_(*t*) at time *t*. From a random walk perspective, if the affect state is located somewhere in this cell at time *t*, it could do five different things within the time interval *Δt*: it could remain there, or it could move to any of its four neighboring cells (diagonal motions are not allowed). The probability of choosing any of these is 1/5. Because the AIM describes a random walk on a potential surface – the free energy function – a move to a neighboring cell, say (*m* − 1,*n*), is only accepted with a probability

$$ k\big[(m,n) \rightarrow (m-1,n)\big] = \min\bigg\{ 1,  e^{F_{m,n} - F_{m-1,n}} \bigg\}, $$

where *F*_*mn*_ denotes the value of the free energy function at the cell center $\left (\frac {2m+1}{2}\delta , \frac {2n+1}{2}\delta \right )$. Hence, if the free energy is lower in the neighboring cell, the update is always accepted (probability 1). If the free energy is higher in the neighboring cell, the update is only accepted with a probability $e^{F_{m,n} - F_{m-1,n}}$. If the state were to choose to remain in the cell (*m*,*n*), the probability of acceptance would also be 1 because the free energy difference between the current cell and the cell it wants to move to is zero – hence, the probability of the state remaining in the current grid cell is at least 1/5; there is a 1/5 chance of selecting this grid cell for the update and the update is accepted with probability 1. If one of the neighboring cells was selected for the update, but the update was rejected, then the state would also remain in the grid cell.

Using this updating scheme, the probability of the affect state moving away from the cell (*m*,*n*) – the out-flux – at time *t* is given by

$$ j^{out}_{m,n}(t) = \frac{1}{5} p_{m,n}(t) \sum\limits_{(m',n') \in \mathcal{N}}  k\big[(m,n) \rightarrow (m',n')\big], $$

where $\mathcal {N} = \big \{ (m-1,n), (m+1,n), (m,n-1), (m,n+1) \big \}$ denotes the set of neighboring cells of (*m*,*n*). The out-flux is equal to the probability of being in the cell (*m*,*n*) times the probability of moving to any of the neighboring cells. In the same vein, the probability of the affect state entering the cell (*m*,*n*) from any of the neighboring cells – the in-flux – at time *t* is given by

$$ j^{in}_{m,n}(t) = \frac{1}{5} \sum\limits_{(m',n') \in \mathcal{N}}  p_{m',n'}(t)  k\big[(m',n') \rightarrow (m,n)\big]. $$

The net flux through the boundary of cell (*m*,*n*) at time *t* is

7 $$ j_{m,n}(t) = j^{in}_{m,n}(t) - j^{out}_{m,n}(t) $$

and thus

8 $$ p_{m,n}(t+{{\varDelta}} t) = p_{m,n}(t) + j_{m,n}(t). $$

### Boundary conditions

At this stage, we have not yet imposed the (no-flux) boundary conditions (5). If we do not do this, probability mass will flow from the interior of the affect space to the exterior. To avoid this, we construct a layer of ghost cells around the interior cells of the affect grid. In a ghost cell, the free energy is infinite. As a consequence, the probability of the affect state moving from a cell on the boundary of the affect grid to a ghost cell is zero – the free energy of an interior cell is always finite. Therefore, the free energy of the ghost cell is infinitely larger than that of the interior cell and the probability of acceptance is zero. If the affect state cannot move from the interior to any of the ghost cells, the flux through the boundary of the affect grid is zero, as required by the boundary conditions.

### The step size

The diffusion constant *D* of the Metropolis-Hastings algorithm described above is defined as

9 $$ D = \frac{\big\langle ({{\varDelta}} y_{i})^{2} \big\rangle}{2 {{\varDelta}} t}. $$

It has the same value for both dimensions *i* = 1 and *i* = 2. This is the same parameter as the time scaling parameter in Eqs. 1 and 3.

Rewriting expression (9), we have that

10 $$ {{\varDelta}} t = \frac{\big\langle ({{\varDelta}} y_{i})^{2} \big\rangle}{2 D}. $$

Assume the affect state is at the site (*m*,*n*). The expected quadratic change along the first dimension is given by

$$ \big\langle ({{\varDelta}} y_{1})^{2} \big\rangle = \frac{\delta^{2}}{5} \bigg(k\big[(m,n) \rightarrow (m-1,n)\big] + k\big[(m,n) \rightarrow (m+1,n)\big] \bigg). $$

Assuming, without loss of generality, that *∂*_1_*F*(**y**) < 0 at the site (*m*,*n*), we have that

$$ k\big[(m,n) \rightarrow (m-1,n)\big] = e^{F_{m,n} - F_{m+1,n}}, $$

and

$$ k\big[(m,n) \rightarrow (m+1,n)\big] = 1. $$

Hence,

$$ \big\langle ({{\varDelta}} y_{1})^{2} \big\rangle = \frac{\delta^{2}}{5} \bigg(e^{F_{m,n} - F_{m+1,n}} + 1 \bigg). $$

Provided the bin width *δ* is small, *F*_*m*,*n*_ − *F*_*m*+ 1,*n*_ will be small and we can use Taylor’s approximation to write

$$ \begin{array}{@{}rcl@{}} e^{F_{m,n} - F_{m+1,n}} &\approx &1 + F_{m,n} - F_{m+1,n} \\ &\approx& 1 - \frac{\partial F(\mathbf{y})}{\partial y_{1}} \delta. \end{array} $$

Using this approximation, the expected quadratic change along the first dimension becomes

$$ \big\langle ({{\varDelta}} y_{1})^{2} \big\rangle = \frac{\delta^{2}}{5} \bigg(2 - \frac{\partial F(\mathbf{y})}{\partial y_{1}} \delta \bigg). $$

Up to second-order in the infinitesimal bin width *δ*, this reduces to

$$ \big\langle ({{\varDelta}} y_{1})^{2} \big\rangle \approx \frac{2\delta^{2}}{5}. $$

A similar derivation for the second dimension would give the same result.

From expression (10), we see that after fixing the bin width *δ* of the grid and after fixing the time scaling parameter *D*, the step size *Δt* of the Metropolis-Hastings updates is fixed at

11 $$ {{\varDelta}} t = \frac{\delta^{2}}{5D}. $$

In other words, the discretization of the affect space and the discretization of time are entwined; the bin width *δ* and the time step *Δt* cannot be specified independently from one another.

### Global minimization

The min-log-likelihood function can have multiple local minima. To find the global minimum, a global optimization heuristic is required. The software package we provide includes an implementation of the differential evolution (DE) global optimization heuristic (Storn and Price, 1997).

Differential evolution is a parallel, stochastic, direct search method for solving continuous optimization problems. It relies on a population of NP model parameter vectors or “agents” which update over generations (iterations). While updating, the population size NP remains constant.

To start a DE optimization, an initial population of agents is sampled from a prior distribution on the parameter space. This prior distribution best covers the entire search space (the part of the parameter space that is of interest). For each of the sampled agents, the min-log-likelihood is computed. This initial population comprises the first generation of agents.

After initialization, the population is updated iteratively. Every population update results in a new generation of agents. The updating of a population happens in three stages. First, for every agent in the population, a mutant vector is constructed by randomly selecting three distinct agents from the population (different from the agent concerned) and adding the weighted difference of two agents to the third. Second, a crossing-over between agents and mutants takes place. During this procedure, the agents’ parameters are intermixed with the parameters of their corresponding mutant vector. The resulting vectors make up the offspring and will be referred to as children. Third, the min-log-likelihood is computed for each of the children. Then, the min-log-likelihood of each child is compared to that of the agent to which it is kin (the parent; i.e., the agent of which it inherited part of its parameters during crossover). If the min-log-likelihood of the child is smaller, it replaces the parent in the population. Otherwise, the parent lives on and the child is disposed of. The remaining agents comprise the next generation.

The summed min-log-likelihood across all agents can only decrease from one generation to the next (DE is a greedy algorithm). Agents that are more optimal (smaller min-log-likelihood) will attract other agents. In doing so, the population increasingly focuses on regions in the parameter space that are more interesting for finding the global minimum. Across generations, the spread of the population naturally shrinks and so do the weighted differences between the agents. Hence, the optimization scheme naturally adapts from that of a global search to that of a local search.

## The software package

We have developed a Julia package (Gradient Diffusion.jl) that enables users to obtain parameter estimates of general bounded drift-diffusion models like the AIM. The package requires users to define a free energy surface *F*(**y**) and functions which appropriately generate and manipulate parameters. These functions have been implemented for the AIM and a bounded version of the OU model, and are readily available in the package. The software package is available at https://ppw.kuleuven.be/okp/software/gradientdiffusion/. We also included documentation, three example data sets (extracts from the original ESM data of Heininga et al., (2019)), and a brief tutorial demonstrating how to use the package.

The package that we provide is actually a synergy of different packages that we developed. Because the DE optimization heuristic is more broadly applicable to general optimization problems, we have written a separate package for this. In a similar vein, the Metropolis-Hastings method that we use to approximate solutions of the Fokker-Planck equation is not specific to the AIM. We therefore isolated this method in a package of its own. The GradientDiffusion.jl package serves as an overarching package which includes all other packages and which provides higher level functions for users to call.

### GPU and CPU

We have implemented the Metropolis-Hastings method both for NVIDIA GPUs and for CPUs. The CPU implementation allows users without an appropriate GPU to use the software package. However, estimations on the CPU are much more time consuming. The GPU implementation is provided to significantly reduce the computational bottleneck of having to compute a tremendous number of conditional probability densities.

#### CUDA kernel

Many conditional probabilities have to be computed in order to update a DE population. These conditional probabilities can be computed in parallel. The computation of a conditional probability density comes down to evaluating numerous updates of grid cells (see the description of the numeric algorithm above). These individual cell updates can also be done largely simultaneously, in parallel.

Much more so than CPUs are GPUs built for massive parallel computations. For that reason, the software package includes a CUDA kernel (written in Julia instead of CUDA C) which computes the numerical solutions to the Fokker-Planck (3) on the GPU. CUDA (Compute Unified Device Architecture) is an extension of the C programming language developed by NVIDIA to optimally program NVIDIA GPUs for custom parallel calculations. By virtue of the GPU, parameter estimates can be obtained much faster than when using a CPU (see benchmarks below).

Because of the specific architecture of NVIDIA GPUs, fixing the size of the affect grids so that they are 30 by 30 (boundary layer of ghost cells excluded) significantly improves the performance. Hence, on the GPU, the bin width is *δ* = 1/30 ≈ 0.033. In principle, bin widths could be made larger or smaller if desired, but because of the computational benefit of using this specific grid size, this option has not been made available for the GPU in the provided package.

#### CPU version of the diffusion kernel

To facilitate distribution of the package, we have also included an implementation of the diffusion kernel which runs entirely on the CPU. Although CUDA is preferred when many data sets or large data sets have to be analyzed (see benchmarks below), the CPU version does allow estimations to be done in the absence of a NVIDIA GPU. However, in order to make estimations feasible on the CPU, we had to rely on the multithreading features of Julia. This means that users can significantly reduce the computational cost of CPU estimations by allowing Julia to use multiple processor cores.

## Benchmarks

We compared the Metropolis-Hastings method to the Euler-Maruyama method for several grid sizes. We also tested the estimation software by means of a recovery study. For both these analyses, we relied on parameter estimates obtained by fitting the AIM to 118 ESM time series concerning the evolution of positive and negative affect. Finally, to give the user an idea of the time it takes to do an estimation, we ran benchmark estimations using three different CPUs and two different GPUs. For these benchmark estimations, the same 118 time series were used.

### Materials and methods

#### Data

The 118 ESM time series were collected in the context of a study regarding symptoms and emotion dynamics in individuals suffering from major depressive disorder and/or borderline personality disorder. An in detail description of the study is given by Heininga et al., (2019). We had no involvement in the study nor in the collection of the data. The study was approved by the Medical Ethics Committee UZ Leuven (B322201627414) and every participant gave informed consent.

Participants were given Motorola Defy Plus Smartphones which they had to carry on them throughout their daily lives. The devices were programmed to send a questionnaire to the participants 10 times a day in between 10 a.m. and 10 p.m. using a stratified random sampling scheme. The time interval between consecutive questionnaires was on average 72 minutes. Each questionnaire consisted of 27 questions, including questions about emotions, social expectancy, emotion regulation, context and psychiatric symptoms. It took participants on average $2'2^{\prime \prime }$ ($SD = 37^{\prime \prime }$) to fill out a questionnaire. The positive (*euphoric*, *happy*, *relaxed*) and negative (*depressed*, *stressed*, *anxious*, *angry*) emotion items could be rated using a continuous slider ranging from 0 (*not at all*) to 100 (*very much*). On average, the participants filled out 87% (*SD* = 11*%*) of the questionnaires that were sent to them. Consequently, the time series on average consisted of 61 actual measurements.

#### Accuracy

It can be shown that the Metropolis-Hastings method discussed in this paper converges to actual results of the Fokker-Planck equation (3) when the bin width *δ* goes to zero (Kikuchi et al., 1991). By comparing the Metropolis-Hastings method to the traditional Euler-Maruyama method, we investigated at what rate the Metropolis-Hastings scheme converges to the actual solutions of the Fokker-Planck equation when the bin width *δ* is reduced. Since we know that the Euler-Maruyama method provides a good approximation of the solutions of the Fokker-Planck equation given an sufficient number of trajectories and a small enough time step, the Euler-Maruyama method is a good reference to study how quickly the Metropolis-Hastings method converges to actual solutions of the Fokker-Planck equation.

To start from relevant AIM parameters, we first fitted the AIM to the 118 time series included in this paper (see Section Data). For each of these 118 parameter sets, we then sampled a random initial condition and compared the probability density conditional on this initial condition obtained with the Metropolis-Hastings method to that obtained with the Euler-Maruyama method at different time intervals.

We considered five different grid sizes: *n* = 15, *n* = 30, *n* = 60, *n* = 120 and *n* = 240. The larger the grid size, the more resolution and the more accurate the approximation should become. For the Euler-Maruyama method, we used 10^7^ trajectories to construct the conditional probability densities. The time step *Δt* of the Euler-Maruyama method was always taken equal to the time step of the Metropolis-Method with the smallest bin width (*δ* = 1/240). We evaluated the conditional probabilities of the two methods after four different time intervals; these intervals were given by $t\frac {(1/30)^{2}}{5D}$ with *t* = 5,50,100,500. Here, *D* represents the (estimated) diffusion constant of the parameter set under consideration. For these time intervals, exactly *t* updates are required for the Metropolis-Hastings algorithm on a 30 × 30 grid (the grid size used for GPU estimations) to obtain the desired conditional probability density. This implies that for larger *t*, the conditional probability density functions lie closer to the equilibrium probability density.

For the sake of comparing the approximations generated by the Euler-Maruyama method and those generated by the Metropolis-Hastings method, we always rebinned the approximations on a 30 × 30 grid. The more bins that are considered, the more trajectories have to be simulated in order to approximate all the bin heights. By always considering the same grid size, and thus the same number of bins, the accuracy of the Euler-Maruyama method was always the same. About 10^4^ trajectories were considered per bin. We compared approximations of the Metropolis-Hastings method with those of the Euler-Maruyama method by taking the *L*^2^-norm of the difference of the approximations on the 30 × 30 grid. We also considered the $L^{\infty }$-norm. The results of this analysis are discussed in Appendix A.

#### Recovery study

With the recovery study, we investigated whether the exact parameters can be retrieved using the estimation procedure in the software package when a sufficient amount of data is simulated. To start from relevant AIM parameters, we first fitted the model to the available 118 data time series (see data section). For each of these 118 parameters sets, we simulated two data sets: one data set with the same sample size as the original observed time series, and another data set with 100 times more data points. For each parameter set, simulated data points were obtained by computing the conditional probability densities starting from the true observations in the original data set and using the true time intervals. Then, respectively 1 and 100 data points were sampled from each of these conditional probability densities. As such, we ensured that data features in the simulated data sets closely matched those of the true data sets. For the first observation of a day, the stationary distribution was used. We used the software package to fit the AIM to every simulated data set and compared the obtained estimates with the corresponding parameters with which the data were simulated.

#### Benchmarks

The estimations were run on three different computing devices, two of which had a NVIDIA GPU. On all devices, the estimations were run using only the CPU (multithreaded on all cores). On the devices including a GPU, the estimations were additionally run using the GPU. The device without GPU was a standard ‘thin and light’ laptop with a IntelⓇ Core^TM^ i7-7600U CPU. The second device was a workstation laptop with a IntelⓇ Core^TM^ i7-9850H CPU and a NVidiaⓇ QuadroⓇ T2000 GPU. The last device we ran the estimations on was a workstation desktop with a IntelⓇ Core^TM^ i7-7800X CPU and a NVidiaⓇ GeForceⓇ RTX^TM^ 2080 Ti GPU. Table 1 lists some of the principal specifications of the three CPUs and Table 2 lists some of the principal specifications of the two GPUs. On all devices, the estimations were done using the single precision floating point format.

**Table 1:** Some CPU specifications

			Frequency		
CPU	Launch year	Cores	Base	Turbo	Vector extensions
			(GHz)	(GHz)	
i7-7600U	2017	2	2.80	3.90	AVX2
i7-9850H	2019	6	2.60	4.60	AVX2
i7-7800X	2017	6	3.50	4.00	AVX-512

**Table 2:** Some CPU specifications

			Frequency		
GPU	Launch year	Memory	Base	Turbo	FP32 performance
		(GB)	(GHz)	(GHz)	(TFLOPS)
T2000	2019	4	1.575	1.785	3.656
RTX 2080 Ti	2018	11	1.350	1.545	13.45

For the estimations, the scores of the positive items and negative items were respectively averaged over to construct positive and negative affect. These were the two variables that we analyzed. To avoid unduly long estimation times, nights were ignored for the estimation procedures – the min-log-likelihood of the first observation after a night was not computed. Although these observations can also be included by evaluating them using the stationary distribution (which is less computationally intensive), we did not do this because we wanted to primarily focus on the computationally intensive aspects of the estimation procedure. Therefore, the very first observation of each time series was not evaluated either, but only used to condition on for the second observation of that day. All estimations on all devices were carried out using the same estimation settings. For the differential evolution, a population size NP of 50 was used and a crossover rate C*R* = 0.6. The number of iterations was fixed at 1000. On every device, only one estimation per time series was run. If an observation was missing, we bridged the gap from the previous measurement to the next. A seed was set before every estimation.

## Results

### Accuracy

**Fig. 2:**
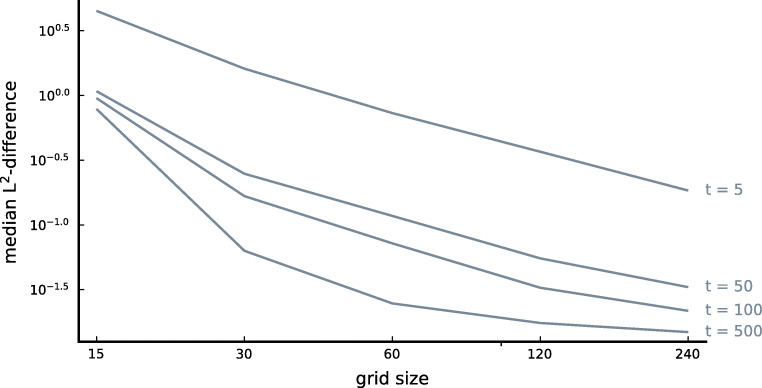
Comparison between the Euler-Maruyama method and the Metropolis-Hastings method The median *L*^2^-difference between the conditional probability densities obtained with the Euler-Maruyama method and those obtained with the Metropolis-Hastings method in function of the grid size. The different lines correspond to different time intervals $t\frac {(1/30)^{2}}{5D}$ (such that for larger time intervals, the conditional distribution lies closer to the equilibrium distribution)

The results of comparing the Metropolis-Hastings method with the Euler-Maruyama method are shown in Fig. 2. In this figure, the median *L*^2^-differences between the conditional probability densities obtained with the Metropolis-Hastings method and those obtained with the Euler-Maruyama method are depicted as a function of the grid size. The different lines correspond to the different time intervals $t\frac {(1/30)^{2}}{5D}$. We see that the conditional probability densities obtained with the Metropolis-Hastings method converge to those obtained with the Euler-Maruyama method when the grid size becomes larger. We also see that differences between the two methods are typically smaller for larger time intervals; the closer the solution to the stationary distribution, the better the approximation – because of the limited resolution of the grid, localized dynamics on shorter time intervals are typically harder to approximate.

### Recovery

**Fig. 3:**
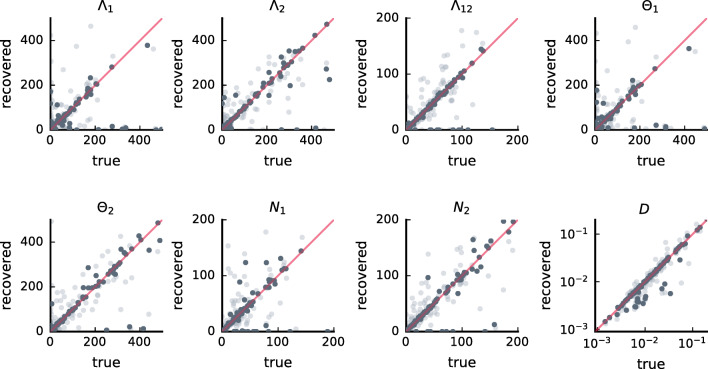
Results of the recovery study. The recovered parameters depicted in function of the true parameters. If the parameter was recovered correctly, the point lies on the main diagonal (depicted in red). Lighter dots correspond to simulated data sets which have the same sample size as the original data sets. Darker dots correspond to data sets with 100 times more data points than the original data sets

Figure 3 depicts the results of the recovery study. Every panel corresponds to a different AIM parameter. In every panel, the estimated parameters are depicted in function of the parameter values that were used to simulate the data. Lighter dots correspond to simulated data sets with the same sample size as the original data sets (about 70 observations) and darker dots correspond to simulated data sets with 100 times more data points. The red diagonal lines indicate where the dots should fall if the parameters with which data was simulated were recovered exactly. For the lighter dots, there is some variability around the diagonals. This is to be expected because of the limited sample sizes of the original data sets (the maximum number of observations was 70). When the sample size becomes larger, parameters are recovered more accurately – the variability of the darker dots around the red diagonal is smaller.

### Benchmarks

**Fig. 4:**
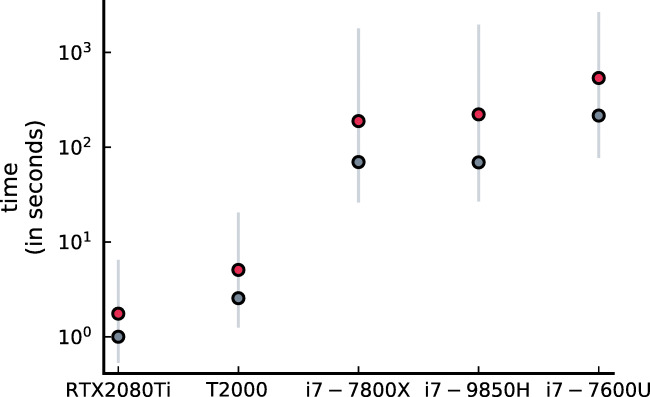
**Benchmarks** The average time per estimation is indicated in red and the median time per estimation is indicated in grey. The grey lines denote the 95% range of the estimation times

The results of the benchmark estimations on the different devices are depicted in Fig. 4. The mean time (in seconds) per estimation is depicted by a red dot. The grey dots indicate the median time per estimation and the lighter lines correspond to the 95% range of the estimation times.

The average time per estimation is 1.75 s for the RTX 2080 Ti GPU, 5.08 s for the T2000 GPU, 188.66 s for the i7-7800X CPU, 222.02 s for the i7-9850H CPU and 537.18 s for the i7-7600U CPU. The slowest GPU (T2000) is on average 37 times faster than the fastest CPU (i7-7800X). For analyzing many and large data sets, it is recommended to use a GPU. Nonetheless, for exploratory analyses, a CPU can be sufficient. A workstation desktop and laptop is still recommended in that case, because they are significantly faster than a standard ‘thin and light’ laptop – for the workstation desktop and laptop it took respectively about 6.2 hours and 7.3 hours to analyze the 118 data sets, while it took 17.6 hours for the standard laptop to finish the same analyses. For the GPUs, it took only a few minutes to do the estimations.

Although the 118 data sets have approximately the same sample size and were obtained in a similar fashion (same sampling schemes with same average time interval), the broad 95% ranges of the estimation times in Fig. 4 indicate that the estimation time is quite variable, even on the same device. This variability has several origins. The sample size determines the number of conditional probability densities that have to be computed in order to compute the min-log-likelihood associated with a specific parameter set and these sample sizes differ. The more observations, the more computations have to be carried out. For the CPU, this is a major concern because it has a limited number of cores to compute the conditional probability densities in parallel. The large number of cores of a GPU allows more conditional probability densities to be computed in parallel, which alleviates the computational burden.

Another important source of variation is the number of steps that typically have to be taken to obtain the numerical conditional probability densities. In Eq. 10 we see that the time step (and thus the number of steps required to bridge a specific time interval) is influenced by the diffusion parameter *D*. This is a parameter that has to be determined during the optimization procedure. Depending on the suggestions and mutations (which are random), parameter values resulting in more steps can occur. Initial *D* values are always sampled from an exponential distribution whose rate parameter has been chosen in such a way that the average number of steps during the initial optimization round equals 25. This small number of steps must ensure that dynamical solutions are visited more frequently in the beginning of the DE optimization than are stationary solutions, since stationary solutions tend to be prominent local attractors. If solutions close to stationarity are more optimal for a specific data set, the number of time steps will gradually increase. For such data sets, estimation times can be a lot longer.

Another source of variation has to do with the inherent randomness of the search algorithm. Search agents are initially sampled randomly from some distribution and are then made to mutate using a scheme that is also inherently random. Hence, analyzing the same data set twice with a different seed will already result in different estimation times, because different parameter sets are considered.

## Conclusion

In this paper, we have introduced numerical tools and an associated (free and open source) Julia software package that can be used to obtain parameter estimates for bounded gradient-drift diffusion models in general. The package comes with an implementation of the nonlinear Affective Ising Model and a bounded version of the Ornstein-Uhlenbeck model (also known as the continuous-time VAR(1) model in the field of affect research).

Because nonlinear models like the AIM typically do not have closed form expressions for the min-log-likelihood function, we relied on a numerical method that could be implemented with a large degree of parallelism. By exploiting this parallelism using Graphics Processing Units, parameter estimates can be obtained in a reasonable amount of time. By comparing the implemented numerical method to the Euler-Maruyama algorithm for different grid resolutions (different bin widths), we demonstrated that both methods converge to the same solutions for sufficiently small time steps and bin widths. We additionally showed that the exact parameters with which data were simulated can be recovered with the estimation software, provided that the sample size of the simulated data set is sufficiently large.

The software package includes functionalities to run the estimations both on GPU and on CPU. The results of the benchmarks indicate that exploratory analyses can be done on CPU but for analyzing many data sets, a GPU is recommended. The GPU devices included in this paper were at least 37 times faster on average than the CPU devices. On average, they are able to finish an estimation in about 1 to 5 seconds.

## Limitations and future directions

The accuracy of the numeric algorithm discussed in this paper depends on the grid resolution (the bin width *δ*). The narrower the bins, the more accurate the numerical solutions to the Fokker-Planck equation, and the more accurate the min-log-likelihood values. Because of the architecture of a GPU, the grid size is fixed at 32 by 32. The outer layer of grid cells are ghost cells used to impose the boundary conditions. As a consequence, the grid size is effectively 30 by 30, and thus the bin width is 0.033. For experience sampling studies where measurements are obtained through self-report, this resolution is likely to be sufficient. Only if individuals are able to distinguish between affect ratings which are less than 0.033 units apart on a zero to one scale, would we have to increase the accuracy of the numerical solutions. Because then, important dynamics could take place on scales smaller than a grid bin and these would go undetected.

In contrast to the GPU, there is no reason to fix the grid size for a CPU. In the Julia package that we provide, the grid size for estimations on the CPU can be adjusted by the user. The default setting is nonetheless to use a 30 × 30 grid, like on the GPU. That way, comparable results can be obtained, irregardless of the hardware that is used (unless the user wants to increase the accuracy); results can differ between the GPU and the CPU because the execution order of specific calculations is not exactly the same on the devices. Another reason not to increase the grid size on the CPU is that CPU estimations are already slower than estimations on the GPU. By increasing the number of bins, more cells have to be updated each Metropolis-Hastings step. Furthermore, because of the relation (11) between the bin width and the step size, the number of Metropolis-Hastings steps required to bridge a specific time interval also significantly increases. It is therefore not recommended to decrease the bin width. Only perhaps to analyze a select number of time series that exhibit very little variability.

Currently, the implementation of the Metropolis-Hastings method that we provide for the GPU only supports NVIDIA GPUs. The reason for this is that we use functionalities that are specific to the CUDA programming language. Since this programming language is specific for NVIDIA GPUs, the implementation is not readily extensible to other GPU devices. In future developments, we may consider adaptations of the implementation which could be extended to other devices.

## Figures and Tables

**Fig. 5:**
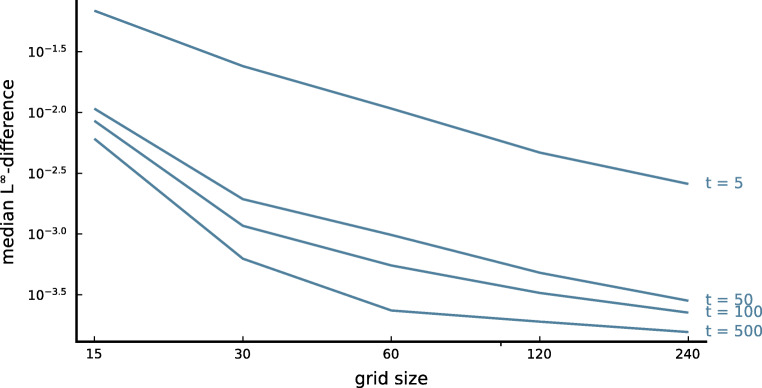
Another comparison between the Euler-Maruyama method and the Metropolis-Hastings method. The median $L^{\infty }$-difference between the conditional probability densities obtained with the Euler-Maruyama method and those obtained with the Metropolis-Hastings method in function of the grid size. The different lines correspond to different time intervals $t\frac {(1/30)^{2}}{5D}$

## Appendix A: No-flux boundary conditions

The Fokker-Planck equation (3) can be rewritten as a continuity equation:

$$ \frac{\partial p(t,\mathbf{y})}{\partial t} - D {\sum}_{i = 1}^{2} \bigg\{ \frac{\partial}{\partial y_{i}}\bigg(\frac{\partial F(\mathbf{y})}{\partial y_{i}} p(t,\mathbf{y}) \bigg) + \frac{\partial^{2}p(t,\mathbf{y})}{\partial {y_{i}^{2}}} \bigg\} = 0. $$

Using vector notation, we can also write this as

$$ \frac{\partial p(t,\mathbf{y})}{\partial t} - D  \nabla \cdot \bigg\{ \nabla F(\mathbf{y}) p(t,\mathbf{y}) + \nabla p(t,\mathbf{y}) \bigg\} = 0. $$

Defining the flux

$$ \mathbf{j}(t,\mathbf{y}) = - D \bigg\{ \nabla F(\mathbf{y}) p(t,\mathbf{y}) + \nabla p(t,\mathbf{y}) \bigg\}, $$

we retrieve a typical continuity equation

$$ \frac{\partial p(t,\mathbf{y})}{\partial t} + \nabla \cdot \mathbf{j}(t,\mathbf{y}) = 0. $$

The vector **j**(*t*,**y**) describes the net probability flux through the point **y** at time *t*. Since there can be no flux through the boundary *∂**Ω* of the affect space, we must have that

$$ \mathbf{j}(t,\mathbf{y}) \bigg\vert_{\partial{{\varOmega}}} = 0, $$

for all *t*. Because *D* is merely a (nonzero) constant, it follows from the definition of the flux that

$$ \bigg\{ \nabla F(\mathbf{y}) p(t,\mathbf{y}) + \nabla p(t,\mathbf{y}) \bigg\} \bigg\vert_{\partial{{\varOmega}}} = 0, $$

or

$$ \bigg\{ \frac{\partial F(\mathbf{y})}{\partial y_{i}} p(t,\mathbf{y}) + \frac{\partial p(t,\mathbf{y})}{\partial y_{i}} \bigg\} \bigg\vert_{\partial{{\varOmega}}} = 0, $$

for all dimensions *i*.

## Appendix B: Comparison of the Euler-Maruyama method and Metropolis-Hastings method: $L^{\infty }$-norm

We also compared the Euler-Maruyama method and the Metropolis-Hastings method using the procedure described in Section Accuracy, but with a $L^{\infty }$-norm instead of a *L*^2^-norm. The results of this comparison are depicted in Fig. 5. In this figure, the median $L^{\infty }$-differences between the conditional probability densities obtained with the Metropolis-Hastings method and those obtained with the Euler-Maruyama method are depicted as a function of the grid size. The different lines correspond to the different time intervals $t\frac {(1/30)^{2}}{5D}$. The figure is comparable to the Fig. 2 for the *L*^2^-norm. We see that the conditional probability densities obtained with the Metropolis-Hastings method converge to those obtained with the Euler-Maruyama method when the grid size becomes larger. We also see that differences between the two methods are typically smaller for larger time intervals; the closer the solution to the stationary distribution, the better the approximation. Because of the limited resolution of the grid, localized dynamics on shorter time intervals are typically harder to approximate.
